# The impact of transcranial direct current stimulation on the neuro-muscular control strategies of penalty kicks in soccer players

**DOI:** 10.3389/fspor.2025.1649809

**Published:** 2025-09-01

**Authors:** Haoyang Wang, Hongxiang Zhang, Xin Li, Jinbang Zhu

**Affiliations:** School of Sport Training, Wuhan Sports University, Wuhan, China

**Keywords:** soccer penalty kick, motor control, muscle synergy, transcranial direct current stimulation (tDCS), non-negative matrix factorization (NMF)

## Abstract

**Objective:**

This study aims to investigate the effects of transcranial direct current stimulation (tDCS) on the neuromuscular control of penalty kicks in soccer players. It also analyzes the relationship between neuromuscular control and the stability of motor performance following the intervention.

**Methods:**

Wireless surface electromyography (EMG) data were synchronously collected from 20 national first-level athletes during penalty kicks using a wireless EMG acquisition device. To obtain spinal-level motor output, the EMG signal envelopes were inversely mapped to the *α*-motor neuron pools across various spinal segments. Muscle synergy characteristics were analyzed using non-negative matrix factorization and K-Means clustering.

**Results:**

During the stance foot contact phase, tDCS led to more concentrated and less variable activation of lower limb muscle synergy, enhancing control of support and force transfer. In the kicking leg swing phase, muscle synergy was activated earlier and decayed more rapidly, maintaining swing velocity and ball striking accuracy while reducing energy expenditure. Additionally, anticipatory synergy adjustments appeared before movement transitions, indicating improved anticipatory action and adjustment capabilities after the intervention. As a result, neuromuscular control optimized the spatiotemporal structure of synergy, improving coordination and yielding more stable penalty kick performance.

**Conclusion:**

Transcranial direct current stimulation can enhance neuromuscular control efficiency by optimizing spinal motor output and improving the spatiotemporal structure of muscle synergy. This results in more stable and effective kicking actions. Reasonably adjusting the timing of tDCS intervention can help improve soccer players’ kicking performance.

## 1 Introduction

In soccer matches, penalty kicks play a crucial role. The implementation and design of all offensive and defensive techniques and tactics ultimately aim to create scoring opportunities and achieve goals (1). Therefore, performance during penalty kicks is a key factor in determining whether a goal is scored (2). Elite athletes can improve the accuracy of penalty kicks by optimizing their motor control strategies (3, 4).

The regulation strategies for penalty actions during a match are relatively diverse. Athletes not only need to maintain a moderately uniform approach speed and stable step frequency (5, 6), but also complete the transition from dynamic running to static kicking (7). This poses significant challenges to neuromuscular regulation and postural stability, requiring the central nervous system (CNS) to coordinate the synergistic activity of multiple muscle groups within a very short time. Under task constraints and biomechanical limitations, the CNS must optimize neuromuscular control strategies. Simultaneously, it needs to integrate peripheral sensory input, continuously evaluate deviations between actual and expected movements, and promptly activate correction mechanisms (8). At present, most research on soccer penalty kicks focuses on the biomechanical characteristics of lower limb joints and muscles (9). Relevant studies have found that high-level soccer players can improve the efficiency of kinetic chain transmission in the swinging leg during the approach by increasing the external rotation angle of the hip joint and enhancing knee joint flexion stiffness in the support leg (7). Li et al. (10) found that elite soccer players facilitate rapid transition of movement states during penalty kicks through pre-activation of the rectus femoris and gluteus maximus muscles in the lower limbs. The specific skills and experience accumulated through long-term training and competition help athletes optimize their neuromuscular control strategies during penalty kicks, thereby enhancing performance.

Transcranial direct current stimulation (tDCS) is a non-invasive neuromodulation technique that modulates the excitability of cortical neurons by applying low-intensity direct current (typically 1–2 mA) to specific brain regions via electrodes placed on the scalp (11). Anodal stimulation enhances neuronal activity and promotes cortical excitability, while cathodal stimulation has the opposite effect (12). In recent years, the application of tDCS in the field of sports science has gradually increased. Studies have shown that tDCS can improve neuromuscular function, enhance motor learning efficiency, and improve muscle coordination by stimulating motor-related brain regions such as the primary motor cortex (M1) (13, 14). Related research also indicates that tDCS has a positive impact on strength output, endurance, motor control, and athletic performance (15, 16). With the gradual application of neuromodulation technologies in elite sports in recent years, tDCS has been widely used to improve soccer players' performance and recovery. In terms of motor skills, Moreira et al. (17) applied anodal tDCS (2 mA, 20 min, for three consecutive days) to the left dorsolateral prefrontal cortex of professional players under simulated fatigue. The results showed significant improvements in response speed and accuracy during passing tasks, as well as enhanced subjective well-being. Regarding cognitive function regulation, Qi et al. (18) stimulated both the motor cortex and the prefrontal cortex under fatigue induction, finding that M1 stimulation improved attention performance. Gonçalves et al. (19) combined tDCS with pneumatic compression therapy in professional male soccer players, and evaluated the intervention using markers such as creatine kinase levels, subjective recovery scores, and delayed-onset muscle soreness. The results showed that the combined intervention effectively reduced muscle damage and improved subjective recovery. Although the above studies have confirmed the potential value of tDCS from performance and cognitive perspectives, further investigation is still needed to explore its specific effects on motor generation mechanisms.

In fact, athletes' biomechanical characteristics reflect the interaction between the nervous system and the external environment under task constraints and biomechanical limitations (20, 21). Therefore, analyzing only the biomechanical characteristics during the penalty phase cannot fully elucidate the specific influence of tDCS on neuromuscular control strategies (22). Thus, it is necessary to further explore the neuromuscular control characteristics of soccer players with different skill levels during penalty kicks under the effect of tDCS.

The central nervous system (CNS) plays a crucial role in coordinating complex multi-muscle motor control and in adapting neuromuscular control strategies during motor skill acquisition (23). Analysis of surface electromyography (sEMG) activity patterns following tDCS application has revealed that tDCS can influence the recruitment of various muscle synergy patterns by modulating motor cortex excitability—selectively enhancing or suppressing muscle groups with specific activation weights or timing characteristics. This suggests that the adjustments in motor control under tDCS are dependent on the dynamic plasticity mechanisms of the neuromuscular system (24). By integrating the effects of neuromodulation with changes in attentional strategies and embedding them into a few coordination modules, it is possible to adapt module parameters to meet specific motor task goals. This enables simplification and efficient modulation of neuromuscular output patterns during the control and optimization of complex kicking actions (25, 26).

Moreover, the neural control mechanism of penalty kicking is the result of complex interactions between supraspinal CNS structures, peripheral sensory feedback, and central pattern generator (CPG) signals (27). The CNS, particularly the cerebral cortex, is responsible for the planning and initiation of penalty kick movements and transmits motor commands to the spinal cord through descending pathways. Subsequently, these commands are further refined at the spinal level, activating specific pools of *α*-motoneurons that drive the contraction of related muscles. Meanwhile, peripheral sensory feedback mechanisms continuously monitor body status and environmental changes, providing essential information to the CNS for real-time adjustment of motor commands. The central pattern generator at the spinal level coordinates the activation sequence and timing of muscles, ensuring movement fluidity and coordination. Ultimately, the activity patterns of *α*-motoneurons at various spinal segments collectively reflect this complex neural control process and directly drive the execution of the penalty kick.

Based on the aforementioned studies on motor control, this study aims to investigate the effects of transcranial direct current stimulation (tDCS) on neuromuscular control strategies during soccer penalty kicks from the perspective of muscle synergies. The study seeks to clarify the physiological significance of muscle coordination during shooting and to analyze the spatiotemporal differences in muscle synergies between athletes of different skill levels under the influence of tDCS.

## 2 Participants and methods

### 2.1 Participants

A total of 20 male national first-class soccer players were recruited as participants in this study. The participants had an average age of 18.52 ± 5.37 years, a height of 178.67 ± 5.52 cm, and a body weight of 70.52±9.65 kg (Detailed information is shown in Table 1: Basic Information of Subjects). All participants were right-leg dominant and had no injuries to key lower limb structures involved in motor performance within six months prior to the test period, in order to eliminate the potential influence of physical injuries on the test results.

**Table 1:** Table of basic information of subjects.

Indicator	Value
Sex/Person	
Male	20.00
Age/years	18.52 ± 5.37
Playing Position/person	
Forward	10.00
Center forward	6.00
Defender	4.00
Years of training (years)	9.75 ± 2.13
Training frequency (sessions·week^−1^)	4.86 ± 0.92
Training duration (minutes·session^−1^)	104.58 ± 14.37

Before the commencement of the experiment, all participants were informed in writing about the potential risks and relevant safety guidelines associated with the test procedures. Written informed consent was obtained from all participants in both Chinese and English prior to their participation. The intervention administered to the participants was transcranial direct current stimulation (tDCS), targeting the motor cortex. Following the stimulation, participants were equipped with wireless surface electromyography (sEMG) devices and performed three penalty kick trials. This study protocol was approved by the Ethics Committee of Wuhan Sports University and complies with the ethical standards described in the Declaration of Helsinki (Approval No. 2025101). Throughout the entire intervention process, none of the participants reported any discomfort or adverse reactions, indicating that the tDCS protocol employed in this study demonstrated good safety and tolerability among young athletes.

### 2.2 Experimental equipment

A 16-channel Delsys wireless surface electromyography (sEMG) system (Delsys TrignoTM, USA; sampling frequency: 2000 Hz) was used to collect electromyographic data. Following established protocols in related studies (28), wireless sEMG sensors were accurately placed on the participants to record muscle activity from 14 muscles involved in the kicking motion of both legs. The selected muscles included: left vastus lateralis, left rectus femoris, left vastus medialis, left biceps femoris, left semitendinosus, left lateral head of the gastrocnemius, right vastus lateralis, right rectus femoris, right vastus medialis, right biceps femoris, right semitendinosus, and right lateral head of the gastrocnemius.Simultaneous video recording was employed to segment the movement phases, ensuring accurate temporal alignment between the sEMG signals and the corresponding motor actions.

Transcranial direct current stimulation (tDCS) was administered using the Halo Sport headset (manufactured in the USA), which contains three foam electrodes with an area of 24 cm^2^ each. The device delivered a 2 mA direct current across the scalp, modulating the excitability of the motor cortices on both sides of the head.

### 2.3 Experimental procedure

Prior to the formal testing, skin preparation was performed on the lower limb muscles involved in the kicking motion. Hair was removed and the skin was cleaned with alcohol before electrode placement. Seven electrodes were affixed to each participant, targeting the following muscles on both legs: vastus lateralis (VL), rectus femoris (RF), vastus medialis (VM), biceps femoris (BF), semitendinosus (ST), tibialis anterior (TA), and the lateral head of the gastrocnemius (GL). Participants then completed a 5 min light jogging warm-up followed by dynamic stretching. After full warm-up, participants performed free practice penalty kicks using their preferred kicking leg to familiarize themselves with the testing procedures. During formal testing, once the flash synchronization signal of the recording system was triggered, all participants used their right leg as the kicking leg and maintained their habitual approach rhythm. Each participant then completed three maximum-effort instep kicks using the medial foot. The number of kicks was consistent before and after the intervention. Wireless surface electromyography (sEMG) data were synchronously recorded from the major lower limb muscle groups, and this phase was segmented accordingly (29).

The tDCS intervention was administered using a transcranial direct current stimulation device. Prior to stimulation, the electrodes were moistened with saline solution to ensure conductivity and proper device function. At the beginning of stimulation, the current intensity was gradually increased over a period of 30 s until reaching 2 mA, and was then maintained at this level for 20 min. Within 5 min after the end of stimulation, participants completed the kicking task to ensure that data collection occurred during the effective period of the tDCS intervention. The safety and feasibility of this specific tDCS stimulation protocol and target region have been validated in previous studies (30, 31).

### 2.4 Movement phase segmentation

**Figure 1:**
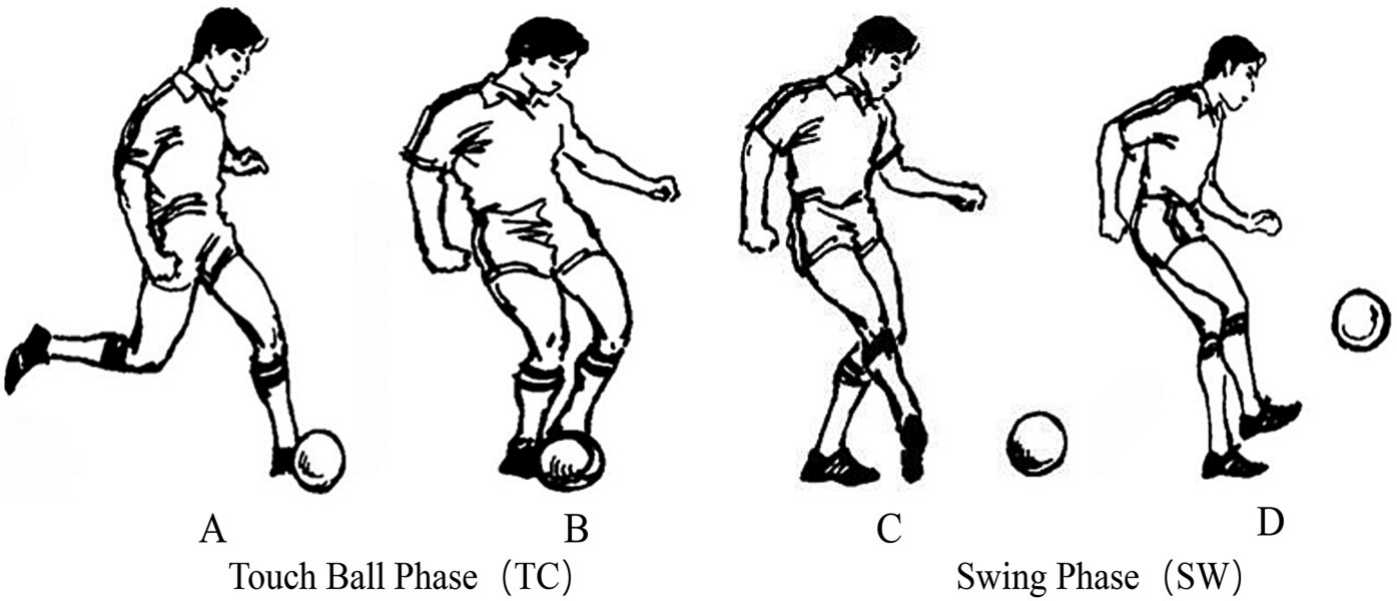
Segmentation of the two phases in penalty kicks. **(A)** Support foot touchdown, **(B)** Ball contact, **(C)** Swing phase in progress, **(D)** End of the kicking phase—segmentation of the Two phases in penalty kicks [adapted from modern soccer (He, 2000)] (37).

Prior to testing, two high-speed cameras were positioned at the front, side, and rear of the testing area, each placed 4 meters from the participant. The optical axes of the two cameras were arranged at a 90-degree angle to each other to ensure comprehensive video capture of the participant's penalty kick motion from multiple perspectives.A frame-by-frame video analysis method was employed (32) to segment the kicking motion. The moment when the supporting foot first made full contact with the ground during the approach run was defined as the support foot touchdown (Figure 1A) (33). The moment when the swinging foot made contact with the ball was defined as the ball contact (Figure 1B). The continuous motion of the swinging foot toward the target was defined as the Swing Phase in progress (Figure 1C), and the moment when the swinging foot landed back on the ground was defined as the end of the kicking phase (Figure 1D) (34). The interval from support foot touchdown to ball contact was defined as the Touch Ball Phase (TC) (35), while the interval from ball contact to the landing of the swinging foot was defined as the Swing Phase (SW) (36). The durations of both the TC and SW phases were time-normalized to 100% (Figure 1).

### 2.5 Data processing

Kinematic and surface electromyography (sEMG) data were preprocessed using MATLAB (R2023a, MathWorks, USA). The raw sEMG signals were sequentially processed using a 50 Hz high-pass filter, full-wave rectification, and a 20 Hz low-pass filter to construct the linear envelope features. All sEMG signals were then normalized to the muscle activation amplitude recorded during the individual's maximum voluntary contraction (MVC) (28).

#### 2.5.1 Spinal segmental motor output

Based on the method proposed by Rabbi et al. (38) and utilizing the myotomal distribution map (39), the sEMG signals from 14 muscles were mapped to the rostral and caudal boundaries of the α-motoneuron (α–MN) pools within the corresponding spinal segments. This approach was employed to evaluate the overall motor output characteristics of the spinal network during the penalty kick, allowing the activation patterns of α–MN pools to be interpreted at the segmental level rather than the individual muscle level. Despite anatomical variability in myotomal distributions across individuals, such differences do not affect the validity of the mapping relationship (40). The motor output pattern of the MNs pool in each active spinal segment *S_j_* during the kicking motion was calculated using the following equation (1):

(1) $$S_{j}=\frac{\sum_{i=1}^{m_{j}}\left(\frac{k_{\;ji}}{n_{{}_{i}}}\times \mathrm{EM}G_{i}\right)}{\sum_{i=1}^{m_{j}}\left(\frac{k_{\;ji}}{n_{i}}\right)}$$

Where, *m_j_* represents the number of muscles innervated by the *j-th* spinal segment, *n_i_* represents the number of spinal segments corresponding to the *i-th* muscle, and *k_ji_* is the weighting coefficient of the *i-th* muscle in the *j-th* spinal segment (41). According to the method of Cappellini et al. (42), the Sj obtained from Equation (1) is multiplied by the number of MNs in each segment (MN*_j_*) to normalize the output.

#### 2.5.2 Muscle synergy extraction

The processed surface electromyography (EMG) signals were subjected to matrix decomposition analysis using the traditional Gaussian Non-negative Matrix Factorization (NMF) method to obtain the characteristic patterns of muscle synergies. According to the research by Esmaeili, S et al. (43) and Cui, C et al. (44), the temporal dynamic features of the synergies are defined as motor primitives, which are the parameters of the temporal activation patterns within the synergy units. The spatial distribution features of the synergies are referred to as motor modules, which are the steady-state muscle group weighting coefficients reflecting the spatial combination patterns within the synergy units. Using NMF, the activation pattern matrix V of the 14 muscles was reconstructed to form *V_r_* (Equation 2) (45):

(2) $$V\approx V_{r}=MP$$

Through Non-negative Matrix Factorization (NMF), the activation pattern matrix V of the 14 muscles is decomposed into *V_r_*, where r represents the number of muscle synergies obtained after decomposition. The matrix M (*m* *×* *r*) is the motor module matrix, which characterizes the relative weights of each muscle in the r synergies. The matrix *P* (*r* *×* *n*) is the motor primitive matrix, which describes the time-varying characteristics of the r synergies. The matrices M and P together characterize the muscle synergies involved in the kicking action. The iterative update of NMF is based on the EM algorithm (Equation 3) (46):

(3) $$\left\{ \begin{array}{c} P_{i+1}=P_{i}\frac{M_{i}^{T}V}{M_{i}^{T}VM_{i}P_{i}}\;\\ M_{i+1}=M_{i}\frac{V{\left(P_{i+1}\right)}^{T}}{M_{i}P_{i+1}{\left(P_{i+1}\right)}^{T}}\; \end{array}\right.$$

According to the research framework of Santuz et al. (45), the convergence condition was set such that the fluctuation of the coefficient of determination R2 between the variable *V* and its reconstructed value *V_r_* was less than 0.01% over 20 consecutive iterations. The quality of the reconstruction from Non-negative Matrix Factorization (NMF) was quantitatively evaluated using Variance Accounted For (VAF) to determine the optimal number of synergies (Equation 4):

(4) $$\mathrm{VAF}=(1\text{-}\frac{{\left\|V-V_{r}\right\|}^{2}}{{\left\|V-\bar{V}\right\|}^{2}})\times 100\text{\%}$$

The Variance Accounted For (VAF) indicates the variance-explaining capability of the reconstructed matrix *V_r_* for the original data matrix *V* when the number of synergies ranges from 1 to 12. By performing linear regression fitting on the relationship curve between VAF and the number of synergies and identifying the point with the greatest change in the slope of the fitted curve (elbow point), the optimal number of synergies is determined. The number of synergies corresponding to this point provides the highest explanation of the original variance with a relatively low number of synergies (47).

The K-Means algorithm was employed to conduct cluster analysis on muscle synergies to obtain the overall synergy characteristics of different groups of athletes and to identify combined synergies. Combined synergies, which are composed of two or more unit synergies, have not yet been fully elucidated in terms of their functional roles and are associated with higher energy metabolism. The proportion of combined synergies in the overall synergy structure can reflect the degree of modular organization of motor commands (48).

In addition, the co-activation index (CI) of antagonistic muscle groups was calculated. The calculation of CI involves the average activity levels of antagonistic muscle groups in the thigh (rectus femoris RF and biceps femoris BF) and the lower leg (medial and lateral gastrocnemius MG-LG and tibialis anterior TA), with the formula as follows (Equation 5) (49, 50):

(5) $$\mathrm{CI}=\frac{1}{200}\sum_{\;j=1}^{200}\left[\frac{EMGH(j)+EMGL(j)}{2}\cdot \frac{EMGL(j)}{EMGL(j)}\right]$$

Here, EMGH and EMGL refer to the activation levels of the muscles with the highest and lowest activation, respectively, within the antagonistic muscle groups after normalization (the EMG activities of the 14 muscles have been normalized to their maximum values). The co-activation index (CI) is calculated as the average of 200 equidistant sampling points within the gait cycle (*j* = 1:200), to obtain the overall co-activation level across the entire movement cycle. This method not only reflects the relative activation intensity of antagonistic muscles at each moment but also reveals the degree of co-contraction across the entire cycle. According to this formula, a higher co-contraction value indicates that both muscles are in a state of strong activation simultaneously, while a lower co-contraction value suggests that the activation of both muscles is weak or that there is a discrepancy between the two, with one being strong and the other weak (51).

### 2.6 Statistical analysis

Prior to data analysis, the Shapiro–Wilk test was used to assess the normality of each variable. If the data met the assumption of normal distribution (i.e., *p* > 0.05), a paired-samples t-test was employed to compare spinal motor output characteristics during penalty kicks among different soccer players after transcranial direct current stimulation (tDCS), as well as to analyze the effects of tDCS on muscle synergies. If the data did not conform to normal distribution, the non-parametric Wilcoxon signed-rank test was used instead. For effect size calculation, Cohen's d was reported when using the t-test, whereas r (Z/√n) was used for non-parametric tests. For each comparison, the *p*-value, effect size, and significance level (α = 0.05) were reported, and all results were summarized in a statistical table for further analysis. All statistical analyses were performed using MATLAB R2024b.

## 3 Results and analysis

### 3.1 Spinal segmental motor output characteristics

**Figure 2:**
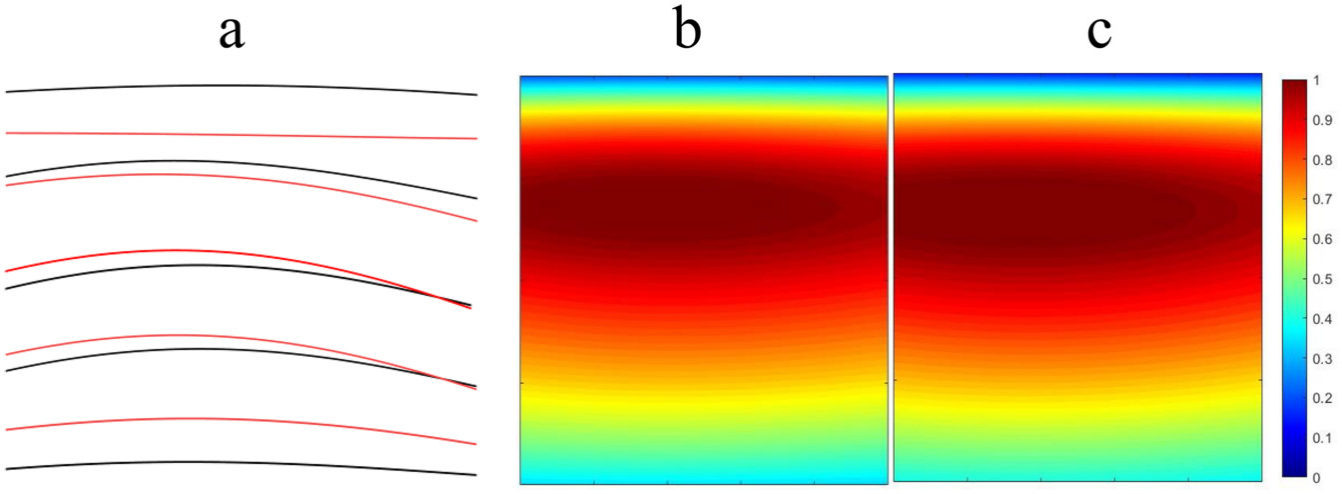
Characteristics of spinal segmental motor output. **(a)** Temporal characteristics of MNs pool output in each spinal segment (black line: before intervention; red line: after intervention); **(b)** Output amplitude of MNs pools in each spinal segment before intervention; **(c)** Output amplitude of MNs pools in each spinal segment after intervention.

Figure 2 illustrates the temporal and spatial distribution characteristics of spinal segment motor output in soccer players before and after the intervention. In Figure 2a, by comparing the temporal characteristics of motor neuron (MNs) pool output in different spinal segments (S3, S2, S1, L5, L4) before and after the intervention (black line and red line, respectively), noticeable changes in the output timing after the intervention can be observed. In particular, segments S1, L5, and L4 exhibit more prominent changes, with the output timing of the L4 segment notably shifted earlier. This may indicate that the intervention had an impact on the activity of motor neurons in these segments.

Figures 2b,c present the output amplitude of MNs pools in each spinal segment before and after the intervention, respectively. Figure 2b shows the output amplitude prior to the intervention, where the color scale from blue to red indicates low to high output amplitude. It can be seen that the output amplitude in segments S3 and S2 is relatively low, while that in S1, L5, and L4 is relatively high. Figure 2c illustrates the post-intervention output amplitude. Compared with Figure 2b, several significant changes are observable. For instance, the output amplitude in the S1 segment increases, with the color changing from blue to yellow, indicating enhanced motor neuron activity in this segment following the intervention. The output amplitude in the L5 and L4 segments demonstrates more complex changes. In the L5 segment, some regions show increased amplitude while others show decreases, suggesting that the intervention may have region-specific effects. The L4 segment shows a general increase in output amplitude, with the color changing from blue to yellow, indicating a significant enhancement of motor neuron activity after the intervention.

In summary, spinal segment motor output in soccer players exhibits significant enhancement in both temporal and spatial characteristics after the intervention, particularly in segments L4 to S1. This may contribute to improved athletic performance and muscle control capabilities.

### 3.2 Muscle co-activation index

**Figure 3:**
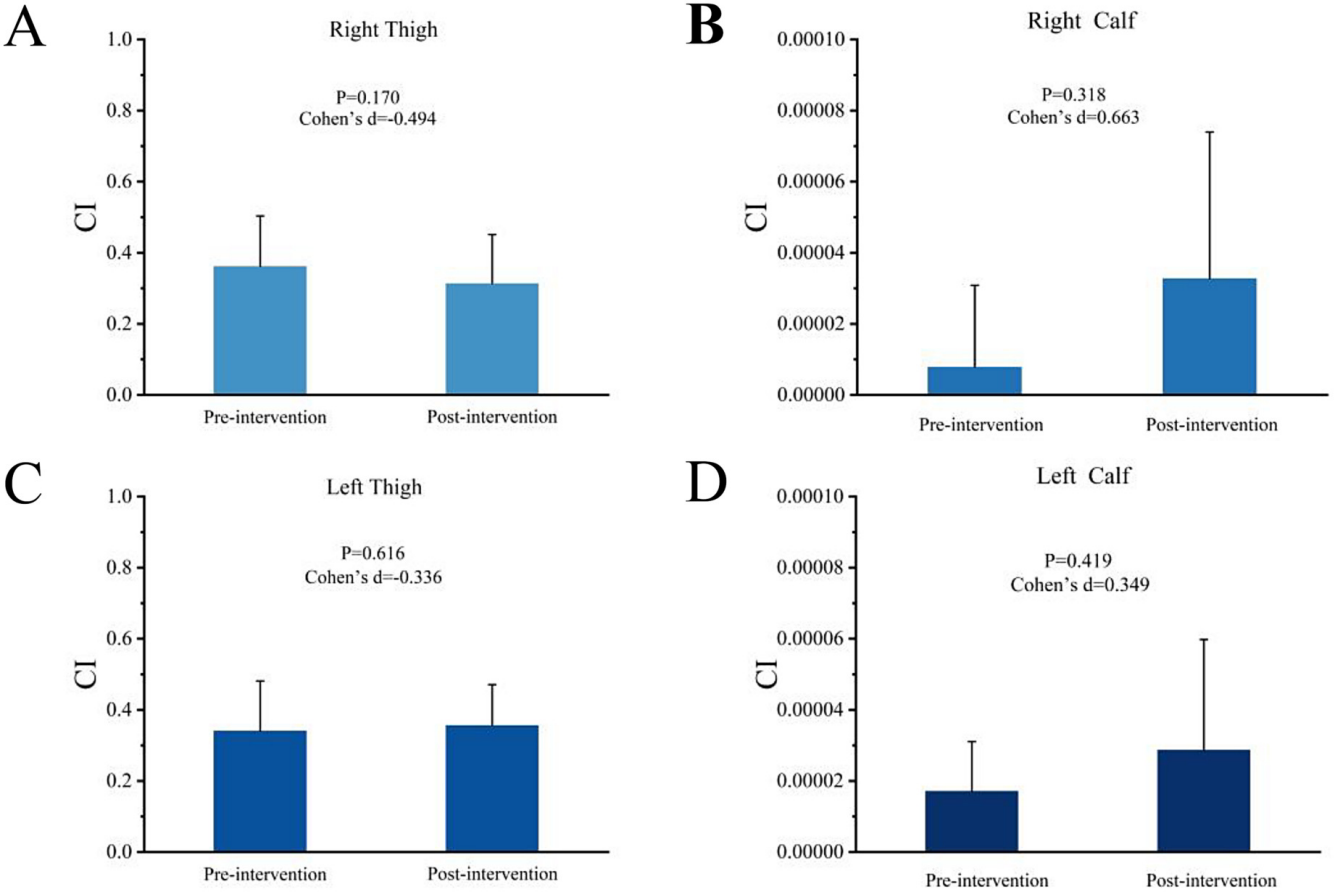
Muscle Co-activation Index. Figure 3 shows the effects of transcranial direct current stimulation (tDCS) on the co-activation index (CI) of 14 muscles in the thighs and lower legs of soccer players. **(A,B)** Light blue bars (Right Thigh and Right Calf) represent the changes in CI values before and after intervention. **(C,D)** Dark blue bars (Left Thigh and Left Calf) represent the changes in CI values before and after intervention. Error bars indicate standard error, but differences before and after intervention did not reach statistical significance.

Figure 3 illustrates the effects of transcranial direct current stimulation (tDCS) intervention on the co-activation index (CI) of 14 muscles in the left and right upper and lower legs of soccer players. As shown in the figure, the CI values of all muscles exhibited changes before and after the intervention; however, these changes did not reach statistical significance (*p* < 0.05). Specifically, the CI values of the right and left thighs slightly decreased after the intervention, while the CI values of the right and left lower legs showed an increase.

### 3.3 Muscle synergy characteristics

**Figure 4:**
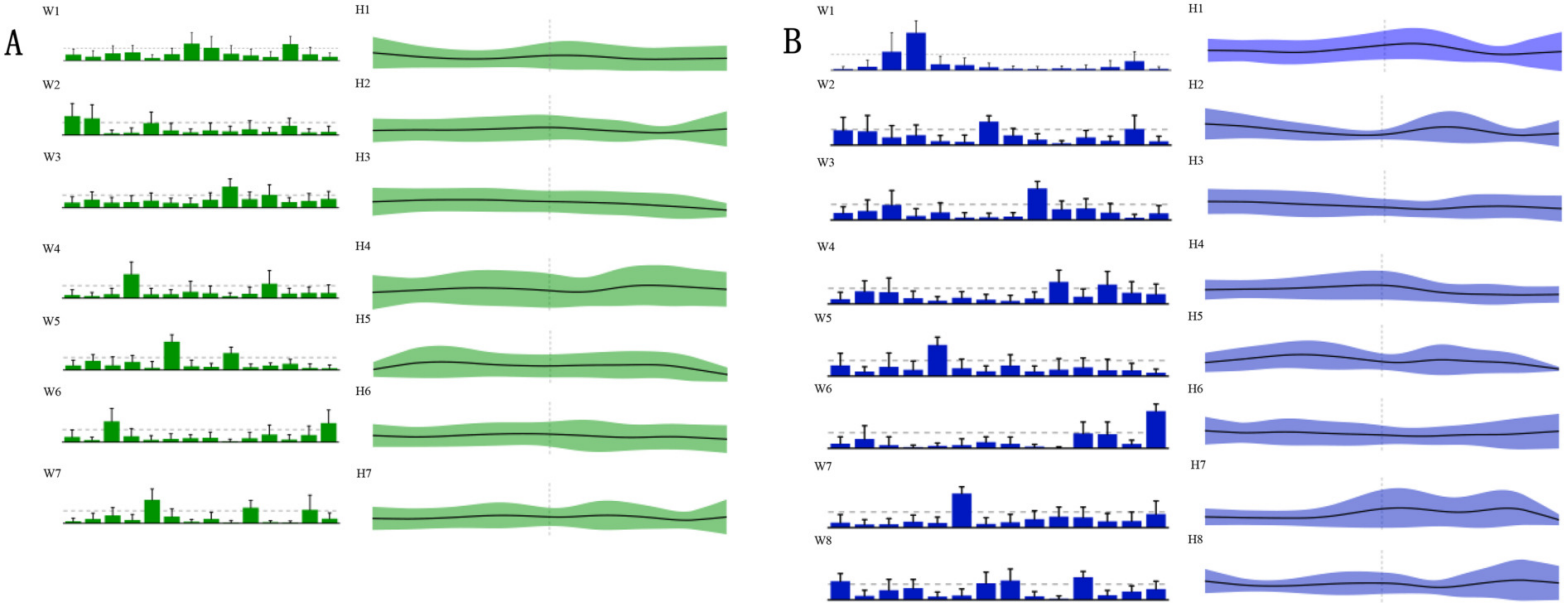
Muscle synergy characteristics. Figure 4 presents the muscle synergy characteristics. The green **A** region denotes the seven motor modules (defined as WSYN1–7) of the pre-intervention muscle synergies; the green H1–H7 denote the motor primitives of the seven pre-intervention muscle synergies. The post-intervention motor modules (defined as SSYN1–8) and the corresponding motor primitives (H1–H8) are shown in the blue **B** region. R-VL, right vastus lateralis; R-RF, right rectus femoris; R-VM, right vastus medialis; R-BF, right biceps femoris; R-ST, right semitendinosus; R-GML, right lateral head of gastrocnemius; L-VL, left vastus lateralis; L-RF, left rectus femoris; L-VM, left vastus medialis; L-BF, left biceps femoris; L-ST, left semitendinosus; L-TA, left tibialis anterior; L-GML, left lateral head of gastrocnemius; R-TA, right tibialis anterior.

This study analyzed the temporal and spatial characteristics of muscle synergies by comparing the penalty kick actions of 20 male national first-class soccer players before and after the intervention. As shown in Figure 4A, the seven muscle synergies (WSYN1–7) exhibited certain regularities in their temporal structure prior to the intervention. The timing and intensity of each motor primitive were evenly distributed across different muscle groups, indicating a natural state of muscle coordination during the penalty kick. Specifically, within WSYN1–7, the activation intensity of the muscle synergies varied over time but remained generally stable overall.

Figure 4B presents the motor modules after the intervention, in which a new synergy group, WSYN8, was introduced, and the motor primitives were extended from H1 to H8. Compared to the pre-intervention state, the post-intervention motor modules showed more pronounced changes in muscle coordination. Notably, in WSYN8, certain muscle groups, such as R-VM and L-VL, exhibited significantly increased activation intensity, indicating a more prominent role of these muscles during the penalty kick. Furthermore, the temporal distribution of the post-intervention motor primitives was more concentrated, reflecting an optimization and adjustment in muscle coordination.

Through the analysis of the muscle synergy characteristics during the penalty kick actions of 20 male national first-class soccer players, it was evident that the intervention had a significant impact on muscle coordination. After the intervention, the players demonstrated a more optimized temporal and spatial distribution of muscle synergies, particularly with enhanced activation intensity in key muscle groups, which may contribute to improved efficiency and accuracy in penalty kicks.

### 3.4 Changes in muscle synergy cluster centers

**Figure 5:**
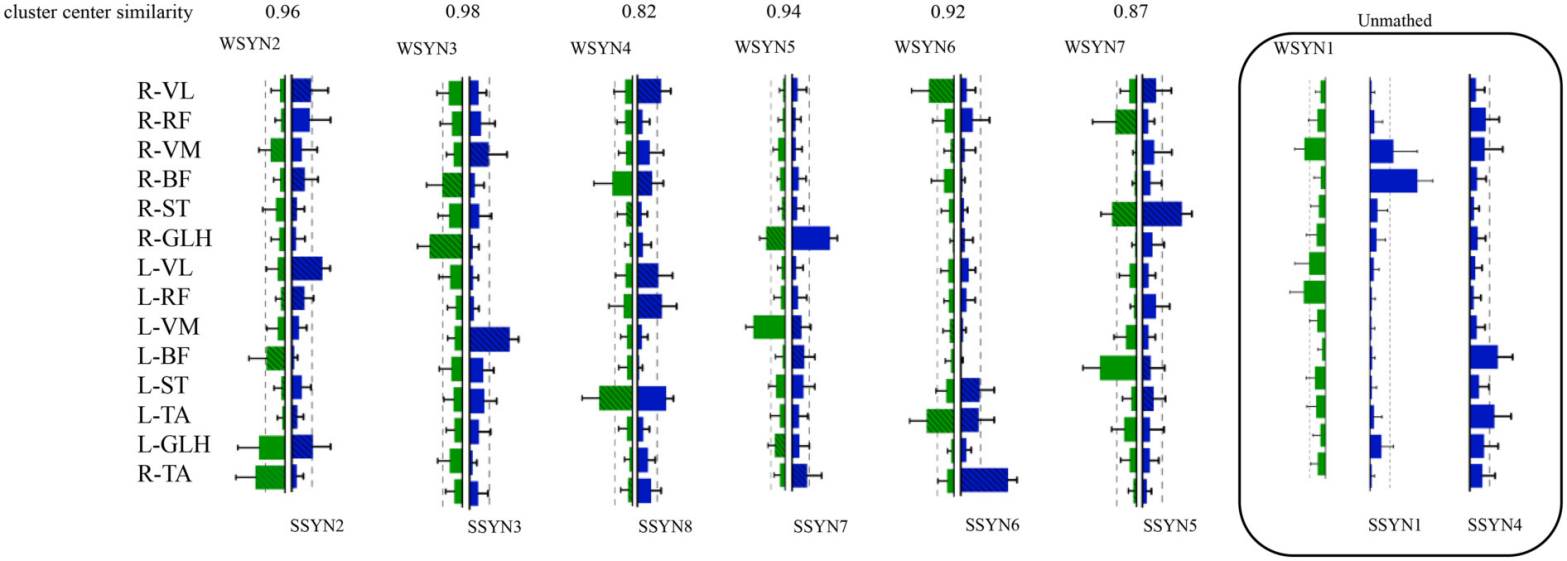
Changes in muscle synergy cluster centers. In Figure 5, the centers of muscle synergies derived from K-means clustering are depicted, with green denoting pre-intervention (six clusters labeled WSYN2–WSYN7) and blue denoting post-intervention (six clusters labeled SSYN2, SSYN3, SSYN5–SSYN8). Cluster matching was performed based on the criterion of maximizing scalar product (i.e., C > 0.8), yielding moderate-to-good correspondence among centers (C ranging from 0.82 to 0.98). The numbers of matched clusters from preparation to completion phases were 4, 3, and 3, respectively. Subject-specific clusters not included in the matching (i.e., synergies contributed by fewer than one-third of the subjects) are indicated by gray shading, namely WSYN1, SSYN1, and SSYN4 (three in total) as shown in the figure. Additionally, the component values of each muscle within every cluster were compared across groups (green and blue with diagonal lines denote *p* < 0.05, paired-samples *t*-test).

Table 2 describes the statistical results before and after the intervention, while Figure 5 illustrates the integration of muscle synergies during the soccer penalty kick, particularly in the TC and SW phases. The figure reports the number (dimensionality) of synergistic activations, the consistency of synergy vectors (C), and the number of clusters determined by the K-means clustering algorithm for each phase.

**Table 2:** Table of statistical results pre- and post-intervention.

Measure	Synergy	muscle	*p*-value	Effect Size (*η²/W*)
		R-VL	0.029*	0.28
		R-RF	0.015*	0.38
		R-VM	0.011*	−0.43
		R-BF	0.012*	−0.74
		R-ST	0.028*	0.63
	WSYN2 VS SSYN2	L-VL	0.024*	−3.43
		L-RF	0.003***	−0.90
		L-BF	0.001***	0.44
		L-ST	0.013*	−0.41
		L-TA	0.011*	0.74
		L-GLH	0.001***	−0.98
		R-TA	0.031*	0.06
		R-VM	0.005***	−0.48
		R-BF	0.034*	0.44
	WSYN3 VS SSYN3	R-GLH	0.041*	0.47
		L-VM	0.045*	−0.47
		R-VL	0.002***	−1.46
		R-BF	0.019*	0.97
		R-ST	0.039*	0.29
FWHM	WSYN4 VS SSYN8	L-VL	0.019*	−0.46
		L-RF	0.002***	−0.42
		L-ST	0.043*	−0.27
		R-TA	0.018*	−0.08
		R-VL	0.039*	0.20
		R-ST	0.025*	−0.13
		R-GLH	0.049*	0.09
		L-VM	0.019*	0.13
	WSYN5 VS SSYN7	L-BF	0.011*	−0.35
		L-GLH	0.031*	−0.11
		R-TA	0.043*	−0.79
		R-VL	0.033*	0.36
		R-VM	0.010*	1.22
		R-GLH	0.018*	1.07
		L-VM	0.011*	−0.02
	WSYN6 VS SSYN6	L-ST	0.014*	−0.57
		L-TA	0.023*	−1.02
		R-TA	0.018*	−1.07
		R-VL	0.029*	−0.81
		R-RF	0.001***	0.19
		R-ST	0.020*	0.20
	WSYN7 VS SSYN5	L-BF	0.017*	0.92
		L-ST	0.030*	−0.80
		R-ST	0.023*	0.57

The results reveal that both phases recruited fourteen muscle synergies. Matched synergies indicate a certain degree of continuity between the TC and SW muscle patterns. Nevertheless, some new synergies emerged in the TC phase, suggesting that increased movement complexity activates additional muscle patterns to accommodate kinematic variations. Across the two phases, partially matched synergies exhibited pronounced differentiation. For example, the broadly distributed synergy in SW—characterized by co-activation of the quadriceps and gastrocnemius—differentiated into more specific functional modules that independently govern hip, knee, and ankle motions. Newly recruited synergies were observed predominantly between these two phases, reflecting heightened neuromuscular control complexity under elevated task demands. From TC to SW, muscle synergies demonstrated dual characteristics of decreasing similarity and activation, alongside differentiation and recruitment. Synergies in the TC phase provided a foundational template for the SW phase, whereas the SW phase adapted to more complex motor requirements through differentiation and recruitment. Compared with TC, the SW phase necessitated a larger number of synergies to coordinate rapid motor demands.

Figure 5 also presents the similarity indices of muscle synergies during the penalty kick. Analyzing the correspondence of synergy cluster centers revealed similarity values ranging from 0.82 to 0.98, indicating that certain neural modules preserved relatively stable muscle coordination across phase transitions. Specifically, WSYN2–SSYN2 (0.96), WSYN3–SSYN3 (0.98), and WSYN5–SSYN7 (0.94) maintained high consistency between phases, suggesting their role as core stabilizing control modules throughout the kick. Conversely, WSYN4 vs. SSYN8 exhibited the lowest similarity (0.82), signifying substantial structural reconfiguration during the transition from TC to SW. Additionally, synergies marked by diagonal shading in the clusters displayed significant changes pre- and post-intervention, particularly involving R-VL, R-ST, L-VL, L-RF, L-ST, and R-TA, indicating that these muscles assumed elevated control loads across the two phases. The distribution of newly recruited synergies overlapped substantially with these altered regions, demonstrating that under increasing task intensity and refined control requirements, the nervous system recruits novel muscle synergy structures to enhance stability and controllability.

In summary, the significant alterations in muscle synergies after intervention indicate differential effects across synergistic modules. While some synergies retained their prominence post-intervention, others underwent change, likely reflecting the intervention's specific influence on muscle synergy patterns.

## 4 Discussion

The present study, from the perspective of spinal segmental output and muscle synergy, provides an in-depth analysis of the effects of transcranial direct-current stimulation (tDCS) on the neuromuscular control strategy underlying the soccer penalty kick. The findings indicate that tDCS not only optimizes the amplitude distribution of spinal *α*-motor-neuron (*α*-MN) pools but also modulates the spatio-temporal structure and coordination degree of lower-limb muscle synergies, thereby promoting movement stability and transition efficiency between the TC and SW phases.

We observed a post-intervention reduction in α-MN pool output at the S3 segment and an increase at L4–S2. Specifically, after the first foot–ground contact of the support leg during the run-up, lower-limb muscles were rapidly recruited with significantly elevated output, resulting in higher ball velocity. In contrast, pre-intervention activation of the lower-limb musculature across L4–S2 was relatively weak. These data suggest that tDCS-mediated optimization of penalty performance is not limited to the visual-aiming skills emphasized in previous studies (52), but also encompasses flexible modulation of lower-limb muscle activation magnitude. Indeed, such modulation is not confined to the penalty task; Zhou et al. (53) demonstrated that elite basketball athletes can redistribute relative activation amplitudes between core and lower-limb muscles during rapid cutting jump-shots to optimize joint loading and improve shooting stability, implying that tDCS optimizes muscle activation strategies mainly by shifting recruitment foci when movement states change rapidly (54, 55). Furthermore, athletes receiving tDCS exhibited significantly elevated muscle activation in the L4–S2 region during the SW phase. The heightened activity of the tibialis anterior and vastus lateralis during this phase may enhance dynamic stability of the ankle and knee joints, thereby facilitating stable foot placement of the support leg. The pronounced output of the long head of the biceps femoris within SW suggests that, post-intervention, athletes achieved more effective hip-extension and knee-flexion control during ball contact. These results likely signify improved sagittal–frontal plane transition capacity and higher efficiency in lower-limb synergistic control. The widespread activation of multiple muscles during SW indirectly corroborates the view that tDCS augments neuromuscular efficiency (56). Nevertheless, quantitative relationships between specific SW-phase muscle activation patterns and penalty accuracy remain to be elucidated.

As the number of penalty trials increases, the quantity of muscle synergies in this complex movement typically rises and may even differentiate into new synergies to accommodate task complexity (57, 58). After tDCS intervention, athletes exhibited a significant increase in the number of synergistic modules compared with pre-intervention values. This outcome suggests that the interaction between neuromuscular regulation and tDCS fosters the emergence of novel synergy modules, thereby enhancing movement flexibility and fine-motor control (59). Correspondingly, post-intervention synergistic activation displayed pronounced phasic and focal characteristics in the temporal domain, whereas the spatial domain was characterized by diverse and complementary co-activation patterns among key muscles. Such synergy expansion likely represents an adaptive mechanism to increased task context complexity and aligns with the notion that “new skill acquisition is accompanied by synergy-system reorganization” (60). Moreover, synergy reconstruction may improve penalty accuracy and consistency, reflected by reduced variability in kinematic parameters (61, 62).

Penalty kicks, as complex motor tasks executed under high-pressure match conditions, demand exquisite neuromuscular coordination. Although previous studies have examined multiple factors influencing the overt features of soccer penalty performance, the neuromuscular control strategies underlying tDCS effects on penalty kicking remain poorly understood (63). In the TC phase, athletes receiving tDCS predominantly recruited lower-limb synergy modules 2 and 4, exhibiting lower activation amplitudes and more concentrated rhythmicity, whereas the non-stimulated group displayed multi-synergy engagement (modules 1, 2, and 4), particularly with pronounced high activation of the rectus femoris and gastrocnemius. This discrepancy likely reflects a more economical control strategy in the tDCS group that balances stability maintenance and force-transmission efficiency (64); conversely, the extensive activation of multiple synergies in the control group may augment energetic cost and muscular redundancy (65), potentially compromising stability of the support phase prior to ball contact (7). During the SW phase, the tDCS group was dominated by synergy module 3 for rapid swing-leg motion, with a shorter activation duration, indicating a refined recruitment strategy after stimulation. In contrast, the control group concurrently recruited synergies 1 and 3, with moderate-to-high activation of the iliopsoas and biceps femoris, revealing large muscular-load discrepancies during the dynamic–static transition. Such inconsistent control patterns may introduce velocity fluctuation at the swing terminus, thereby degrading accuracy and consistency (66). Thus, tDCS can enhance dynamic coherence and movement efficiency between support and swing phases by optimizing the synergistic control architecture of lower-limb muscles.

Across TC and SW phases, the stimulated group exhibited more concentrated and sequential synergy activation, further supporting previous findings that tDCS improves lower-limb neural control efficiency (67). In TC, stability and force-path optimization were primarily achieved via synergies 2 and 4, whereas in SW, rapid swing and ball control were balanced by the early initiation and rapid decay of synergy 3. By contrast, the control group displayed frequent synergy switching and large activation fluctuations during TC, potentially compromising control consistency and energetic efficiency (68). Furthermore, synergy indices declined toward the end of SW, indicating attenuated inter-synergy coordination characteristic of anticipatory synergy adjustments (ASAs) (69). In the stimulated group, ASAs appeared earlier and changed more rapidly, suggesting that tDCS enhanced athletes' anticipatory capacity regarding impending action outcomes, thereby facilitating temporal control and stability during penalty execution (70). These data indicate that neural modulation not only optimized the internal activation structure of individual synergies but also improved inter-synergy coordination strategies, ultimately enhancing stability and accuracy of the penalty kick (71).

Although the muscle co-activation index (CI) did not differ statistically, the post-intervention upward trend in CI values for the lower-leg musculature may imply tDCS-mediated modulation of joint stability. Such fine-tuning between force output and movement control provides a reference for future investigations into the relationship between synergy efficiency and movement precision. Synergy analysis revealed an increased number of synergy modules and more focal temporal structures after tDCS, accompanied by enhanced activation of key muscles such as R-VM and L-VL, indicating more refined and efficient control programming. Clustering of synergies demonstrated a transition from a general template in TC to task-specific patterns in SW; the overlap between newly recruited synergies and key muscle regions suggests that tDCS facilitated adaptive reorganization of the nervous system to complex movements. This synergy reconstruction not only enhances penalty stability and consistency but also underscores the potential of tDCS for optimizing muscle coordination mechanisms.

In the present study, to explore the influence of transcranial direct-current stimulation (tDCS) on the neuromuscular control strategies of soccer players during penalty kicks, we adopted a single-subject pre–post design without a control group. Although most tDCS investigations employ double-blind, crossover, or sham-stimulation controls to strengthen internal validity, studies using single-subject, no-control designs exist. For instance, Rembrandt and Riley (72), in a pilot study, applied a single-session prefrontal tDCS without a control group and observed immediate post-stimulus changes in cortical activity, demonstrating that even in the absence of sham control, tDCS effects on cortical activity can be revealed. This paradigm provided methodological guidance for the current study, especially in skill-oriented motor neuroscience contexts. Nevertheless, the present design carries several non-negligible limitations. The absence of a sham-stimulation control prevents the complete exclusion of placebo effects, learning effects, or other non-specific changes attributable to attention alone, thereby weakening causal inference. Specifically, the lack of a control group may lead to either overestimation or underestimation of the true intervention effects. In the absence of a control condition, observed improvements may be attributable to psychological factors—such as placebo effects or participants' belief in the intervention (73)—rather than to genuine physiological or neural changes. In addition, the possibility of learning effects cannot be ruled out, whereby participants may improve simply through repeated exposure to the task (74). In addition, the possibility of learning effects cannot be ruled out, whereby participants may improve simply through repeated exposure to the task (75). This issue is particularly relevant in complex motor tasks such as soccer penalty kicks, where repeated trials may facilitate skill refinement even in the absence of direct stimulation effects.Moreover, single-session effects are often subject to substantial inter-individual variability, which may be influenced by baseline status, psychological motivation, circadian rhythms, and other factors—an issue particularly pronounced in small-sample studies. For instance, recent research on tDCS in sports has emphasized that individual variability is a critical consideration, with some athletes showing stronger responses to tDCS than others (76). Furthermore, as the present study focuses on the soccer penalty kick—a complex motor behavior involving coordinated regulation across multiple cortical and spinal mechanisms—the response observed in a single subject may not be generalizable to a broader athletic population. To enhance interpretive power, future research should aim to validate these preliminary findings with larger samples, randomized controlled designs, and multiple stimulation sessions. Future tDCS studies could further prioritize task-specific neural modulation characteristics by selecting individualized stimulation sites and parameter configurations to improve targeting and efficacy. The concurrent application of multimodal techniques, such as neuroimaging and surface electromyography, could facilitate the development of quantifiable neuro–behavioral linkage models, thereby elucidating causal pathways between stimulation mechanisms and performance enhancement and exploring potential synergistic effects with skill training, cognitive training, or other interventions. Advancing these research directions is expected to provide a more robust theoretical and empirical foundation for the scientific application of transcranial direct-current stimulation (tDCS) in competitive sports.

## 5 Conclusion

Transcranial direct current stimulation (tDCS) an optimize the spatio-temporal structure of spinal motor output and muscle synergies, enhance neuromuscular control efficiency, and thereby improve penalty-kick stability; appropriately adjusting the timing of transcranial direct-current stimulation intervention can facilitate improved penalty performance in soccer players.

## Data Availability

The datasets presented in this article are not readily available because they are subject to a collaboration agreement and may not be disclosed without authorization; members of the research institution may only access them under a confidentiality agreement. Requests to access the datasets should be directed to wanghy9910@gmail.com.

## References

[1] Loutfi I Gómez-Jordana LI Ric A Milho J Passos P. Highlighting shooting opportunities in football. Sensors. (2023) 23(9):4244. 10.3390/s23094244 37177448 PMC10181708

[2] van Hemert R van der Kamp J Hartman E. The influence of situational constraints on in-game penalty kicks in soccer. Int J Perform Anal Sport. (2024) 0(0):1–13. 10.1080/24748668.2024.2430840

[3] Prieto-Lage I Argibay-González JC Bezerra P Cidre-Fuentes P Reguera-López-de-la-Osa X Gutiérrez-Santiago A. Analysis of penalty kick performance in the Spanish football league: a longitudinal study. Appl Sci. (2024) 14(16):7046. 10.3390/app14167046

[4] Bloechle JL Audiffren J Naour TL Alli A Simoni D Wüthrich G It’s not all in your feet: improving penalty kick performance with human-avatar interaction and machine learning. Innovation. (2024) 5(2):100584. 10.1016/j.xinn.2024.100584 38445019 PMC10912701

[5] Barbero JR Ajamil DL Arana J Anguera MT. Sequential analysis of the interaction between kicker and goalkeeper in penalty kicks. Cuad Psicol Deporte. (2024) 24(3):208–24. 10.6018/cpd.622011

[6] Pinheiro G Feber P. Strategic insights into penalty kick execution: a comprehensive analysis of observable behaviors in the German Bundesliga. Turk J Kinesiol. (2024) 10(2):79–100. 10.31459/turkjkin.1459430

[7] Zhang Y Navandar A Navarro E. The role of support leg kinematics in ball velocity and spin across competitive levels and leg preference. Appl Sci. (2025) 15(12):6473. 10.3390/app15126473

[8] Hewitson CL Kaplan DM Crossley MJ. Error-independent effect of sensory uncertainty on motor learning when both feedforward and feedback control processes are engaged. PLoS Comput Biol. (2023) 19(9):e1010526. 10.1371/journal.pcbi.1010526 37683013 PMC10522034

[9] Doewes R. Different biomechanics in football shooting using inside and instep kick. Int J Disabil Sports Health Sci. (2023) 6(3):307–15. 10.33438/ijdshs.1290078

[10] Li D Elbanna H Lin FY Lu CJ Chen LJ Lu G Neuromotor mechanisms of successful football penalty kicking: an EEG pilot study. Front Psychol. (2025) 16:452443. 10.3389/fpsyg.2025.1452443 PMC12127302 40458627

[11] Nitsche MA Paulus W. Excitability changes induced in the human motor cortex by weak transcranial direct current stimulation. J Physiol. (2000) 527(3):633–9. 10.1111/j.1469-7793.2000.t01-1-00633.x 10990547 PMC2270099

[12] Stagg CJ Nitsche MA. Physiological basis of transcranial direct current stimulation. Neuroscientist. (2011) 17(1):37–53. 10.1177/1073858410386614 21343407

[13] Qi S Cao L Wang Q Sheng Y Yu J Liang Z. The physiological mechanisms of transcranial direct current stimulation to enhance motor performance: a narrative review. Biology. (2024) 13(10):790. 10.3390/biology13100790 39452099 PMC11504865

[14] Rizvi A Bell K Yang D Montenegro MP Kim H Bao S Effects of transcranial direct current stimulation over human motor cortex on cognitive-motor and sensory-motor functions. Sci Rep. (2023) 13(1):20968. 10.1038/s41598-023-48070-z 38017091 PMC10684512

[15] Pallanti S Colzi C Pallanti S Colzi C. Transcranial direct current stimulation (tDCS) on soccer players: a mini-review. Arch Sports Med Physiother. (2024) 9(1):001–7. 10.17352/asmp.000019

[16] Yu Y Zhang X Nitsche MA Vicario CM Qi F. Does a single session of transcranial direct current stimulation enhance both physical and psychological performance in national- or international-level athletes? A systematic review. Front Physiol. (2024) 15:1365530. 10.3389/fphys.2024.1365530 38962069 PMC11220198

[17] Shiravand F Motamedi P Amani-Shalamzari S Amiri E da Silva Machado DG. Effect of repeated sessions of transcranial direct current stimulation on subjective and objective measures of recovery and performance in soccer players following a soccer match simulation. Sci Rep. (2024) 14(1):20809. 10.1038/s41598-024-71701-y 39242725 PMC11379740

[18] Qi F Zhang N Nitsche MA Yi L Zhang Y Yue T. Effects of dual-site anodal transcranial direct current stimulation on attention, decision-making, and working memory during sports fatigue in elite soccer athletes. J Integr Neurosci. (2025) 24(1):26401. 10.31083/JIN26401 39862014

[19] Gonçalves DS Moscaleski LA da Silva GM Morgans R Okano AH Moreira A. The effect of combined transcranial direct current stimulation and pneumatic compression as part of a comprehensive recovery strategy in professional male top-level soccer players. J Strength Cond Res. (2024) 38(9):1658. 10.1519/JSC.0000000000004844 39074250

[20] Dury J Sagawa Y Michel F Ravier G. Neuromuscular fatigue and cognitive constraints independently modify lower extremity landing biomechanics in healthy and chronic ankle instability individuals. J Sports Sci. (2024) 42(14):1341–54. 10.1080/02640414.2024.2391209 39136418

[21] Mulla DM Keir PJ. Neuromuscular control: from a biomechanist’s perspective. Front Sports Act Living. (2023) 5:1217009. 10.3389/fspor.2023.121700 37476161 PMC10355330

[22] Secco Faquin B Teixeira LA Coelho Candido CR Boari Coelho D Bayeux Dascal J Alves Okazaki VH. Prediction of ball direction in soccer penalty through kinematic analysis of the kicker. J Sports Sci. (2023) 41(7):668–76. 10.1080/02640414.2023.2232679 37409691

[23] Herzog M Krafft FC Fiedler J Berger DJ Sloot LH d’Avella A The central nervous system adjusts muscle synergy structure and tightly controls rollator-supported transitions between sitting and standing. J NeuroEngineering Rehabil. (2025) 22(1):96. 10.1186/s12984-025-01622-y PMC12032710 40281643

[24] Chen S Luo Z Lai J. Influence of anodal tDCS on the brain functional networks and muscle synergy of hand movements. J Integr Neurosci. (2024) 23(1):22. 10.31083/j.jin2301022 38287857

[25] Siddhad G Singh J Roy PP. Mecasa: Motor execution classification using additive self-attention for hybrid EEG-fNIRS data. *arXiv* [Preprint]. *arXiv:2501.05525 * (2025). Available online at: http://arxiv.org/abs/2501.05525 (Accessed August 12, 2025).

[26] Kamat A Rahul R Dutta A Cavuoto L Kruger U Burke H Dynamic directed functional connectivity as a neural biomarker for objective motor skill assessment. *arXiv* [Preprint]. *arXiv:2502.13362 * (2025). Available online at: http://arxiv.org/abs/2502.13362 (Accessed August 12, 2025).

[27] Avaltroni P Cappellini G Sylos-Labini F Ivanenko Y Lacquaniti F. Spinal maps of motoneuron activity during human locomotion: neuromechanical considerations. Front Physiol. (2024) 15:1389436. 10.3389/fphys.2024.1389436 39108539 PMC11300930

[28] Hermens HJ Freriks B Disselhorst-Klug C Rau G. Development of recommendations for SEMG sensors and sensor placement procedures. J Electromyogr Kinesiol. (2000) 10(5):361–74. 10.1016/S1050-6411(00)00027-4 11018445

[29] Lees A Asai T Andersen TB Nunome H Sterzing T. The biomechanics of kicking in soccer: a review. J Sports Sci. (2010) 28(8):805–17. 10.1080/02640414.2010.481305 20509089

[30] Okano AH Fontes EB Montenegro RA de Farinatti PTV Cyrino ES Li LM Brain stimulation modulates the autonomic nervous system, rating of perceived exertion and performance during maximal exercise. Br J Sports Med. (2015) 49(18):1213–8. 10.1136/bjsports-2012-091658 23446641

[31] Bikson M Grossman P Thomas C Zannou AL Jiang J Adnan T Safety of transcranial direct current stimulation: evidence based update 2016. Brain Stimul Basic Transl Clin Res Neuromodulation. (2016) 9(5):641–61. 10.1016/j.brs.2016.06.004 PMC5007190 27372845

[32] Nunome H IkegamI Y Kozakai R Apriantono T Sano S. Segmental dynamics of soccer instep kicking with the preferred and non-preferred leg. J Sports Sci. (2006) 24(5):529–41. 10.1080/02640410500298024 16608767

[33] Rabello R Bertozzi F Galli M Zago M Sforza C. Lower limbs muscle activation during instep kick in soccer: effects of dominance and ball condition. Sci Med Footb. (2022) 6(1):40–8. 10.1080/24733938.2021.1884283 35236218

[34] Jönhagen S Ericson MO Németh G Eriksson E. Amplitude and timing of electromyographic activity during sprinting. Scand J Med Sci Sports. (1996) 6(1):15–21. 10.1111/j.1600-0838.1996.tb00064.x 8680937

[35] Nunome H Asai T Ikegami Y Sakurai S. Three-dimensional kinetic analysis of side-foot and instep soccer kicks. Med Sci Sports Exerc. (2002) 34(12):2028. 10.1097/00005768-200212000-00025 12471312

[36] Katis A Giannadakis E Kannas T Amiridis I Kellis E Lees A. Mechanisms that influence accuracy of the soccer kick. J Electromyogr Kinesiol. (2013) 23(1):125–31. 10.1016/j.jelekin.2012.08.020 23021602

[37] Zhilin H. Modern Football*. Beijing: People’s Sports Publishing House (2000).

[38] Rabbi MF Pizzolato C Lloyd DG Carty CP Devaprakash D Diamond LE. Non-negative matrix factorisation is the most appropriate method for extraction of muscle synergies in walking and running. Sci Rep. (2020) 10(1):8266. 10.1038/s41598-020-65257-w 32427881 PMC7237673

[39] Kendall FP Kendall FP. editors. Muscles: Testing and Function with Posture and Pain. 5th ed. Baltimore, MD: Lippincott Williams & Wilkins (2005). p. 480.

[40] Cappellini G Ivanenko YP Dominici N Poppele RE Lacquaniti F. Migration of motor pool activity in the spinal cord reflects body mechanics in human locomotion. J Neurophysiol. (2010) 104(6):3064–73. 10.1152/jn.00318.2010 20881204

[41] La Scaleia V Ivanenko YP Zelik KE Lacquaniti F. Spinal motor outputs during step-to-step transitions of diverse human gaits. Front Hum Neurosci. (2014) 8:305. 10.3389/fnhum.2014.00305 24860484 PMC4030139

[42] Cappellini G Ivanenko YP Martino G MacLellan MJ Sacco A Morelli D Immature spinal locomotor output in children with cerebral palsy. Front Physiol. (2016) 7:478. 10.3389/fphys.2016.00478 27826251 PMC5078720

[43] Esmaeili S Karami H Baniasad M Shojaeefard M Farahmand F. The association between motor modules and movement primitives of gait: a muscle and kinematic synergy study. J Biomech. (2022) 134:110997. 10.1016/j.jbiomech.2022.110997 35219145

[44] Caihong CUI Huacong M Tie L Xiuling LIU Xiaoguang LIU. Analysis of muscle synergy and muscle functional network at different walking speeds based on surface electromyographic signal. J Biomed Eng. (2023) 40:938–44. 10.7507/1001-5515.202303065 PMC10600430 37879923

[45] Santuz A Ekizos A Janshen L Baltzopoulos V Arampatzis A. The influence of footwear on the modular organization of running. Front Physiol. (2017) 8:958. 10.3389/fphys.2017.00958 29213246 PMC5702634

[46] Lee D Seung HS. Algorithms for non-negative matrix factorization. In: Leen TK, Dietterich TG, Tresp V, editors. Advances in Neural Information Processing Systems. Cambridge, MA: MIT Press (2000). p. 556–62. Available online at: https://proceedings.neurips.cc/paper_files/paper/2000/hash/f9d1152547c0bde01830b7e8bd60024c-Abstract.html (Accessed August 12, 2025).

[47] Ballarini R Ghislieri M Knaflitz M Agostini V. An algorithm for choosing the optimal number of muscle synergies during walking. Sensors. (2021) 21(10):3311. 10.3390/s21103311 34064615 PMC8151057

[48] d’Avella A Bizzi E. Shared and specific muscle synergies in natural motor behaviors. Proc Natl Acad Sci. (2005) 102(8):3076–81. 10.1073/pnas.0500199102 15708969 PMC549495

[49] Ervilha UF Graven-Nielsen T Duarte M. A simple test of muscle coactivation estimation using electromyography. Braz J Med Biol Res. (2012) 45:977–81. 10.1590/S0100-879X2012007500092 22641413 PMC3854180

[50] Boudarham J Hameau S Zory R Hardy A Bensmail D Roche N. Coactivation of lower limb muscles during gait in patients with multiple sclerosis. PLoS One. (2016) 11(6):e0158267. 10.1371/journal.pone.0158267 27336442 PMC4919099

[51] Gagnat Y Brændvik SM Roeleveld K. Surface electromyography normalization affects the interpretation of muscle activity and coactivation in children with cerebral palsy during walking. Front Neurol. (2020) 11:202. 10.3389/fneur.2020.00202 32362862 PMC7180206

[52] Zachry T Wulf G Mercer J Bezodis N. Increased movement accuracy and reduced EMG activity as the result of adopting an external focus of attention. Brain Res Bull. (2005) 67(4):304–9. 10.1016/j.brainresbull.2005.06.035 16182938

[53] Zhou Q Wu S Zhang J Pan Z Kang Z Ma Y. Research on the impact of shot selection on neuromuscular control strategies during basketball shooting. Sensors. (2025) 25(13):4104. 10.3390/s25134104 40648359 PMC12251698

[54] Angius L Ansdell P Škarabot J Goodall S Thomas K Cowper G Anodal tDCS improves neuromuscular adaptations to short-term resistance training of the knee extensors in healthy individuals. J Neurophysiol. (2024) 132(6):1793–804. 10.1152/jn.00289.2024 39475491 PMC11687829

[55] Moshashaei MS Gandomi F Amiri E Maffulli N. Anodal tDCS improves the effect of neuromuscular training on the feedforward activity of lower extremity muscles in female taekwondo athletes with dynamic knee valgus. Sci Rep. (2024) 14(1):20007. 10.1038/s41598-024-70328-3 39198471 PMC11358470

[56] Vargas VZ Baptista AF Pereira GOC Pochini AC Ejnisman B Santos MB Modulation of isometric quadriceps strength in soccer players with transcranial direct current stimulation: a crossover study. J Strength Cond Res. (2018) 32(5):1336. 10.1519/JSC.0000000000001985 28489629

[57] Safavynia SA Ting LH. Task-level feedback can explain temporal recruitment of spatially fixed muscle synergies throughout postural perturbations. J Neurophysiol. (2012) 107(1):159–77. 10.1152/jn.00653.2011 21957219 PMC3349688

[58] Dominici N Ivanenko YP Cappellini G d’Avella A Mondì V Cicchese M Locomotor primitives in newborn babies and their development. Science. (2011) 334(6058):997–9. 10.1126/science.1210617 22096202

[59] Waters-Metenier S Husain M Wiestler T Diedrichsen J. Bihemispheric transcranial direct current stimulation enhances effector-independent representations of motor synergy and sequence learning. J Neurosci. (2014) 34(3):1037–50. 10.1523/JNEUROSCI.2282-13.2014 24431461 PMC3891947

[60] Park S Caldwell GE. Muscle synergies are modified with improved task performance in skill learning. Hum Mov Sci. (2022) 83:102946. 10.1016/j.humov.2022.102946 35334208

[61] Torbati AHM Gholibeigi M Jami S. New method to assess and enhance Athletes’ performance based on muscle synergy patterns: a new approach to design a biofeedback training scheme. Eur J Sport Sci. (2022) 1(5):8–14. 10.24018/ejsport.2022.1.5.32

[62] Tajik R Dhahbi W Fadaei H Mimar R. Muscle synergy analysis during badminton forehand overhead smash: integrating electromyography and musculoskeletal modeling. Front Sports Act Living. (2025) 7:1596670. 10.3389/fspor.2025.1596670 40529409 PMC12170632

[63] Scurr JC Abbott V Ball N. Quadriceps EMG muscle activation during accurate soccer instep kicking. J Sports Sci. (2011) 29(3):247–51. 10.1080/02640414.2010.523085 21170796

[64] van Asseldonk EHF Boonstra TA. Transcranial direct current stimulation of the leg motor Cortex enhances coordinated motor output during walking with a large inter-individual variability. Brain Stimul Basic Transl Clin Res Neuromodulation. (2016) 9(2):182–90. 10.1016/j.brs.2015.10.001 26553475

[65] Inouye JM Valero-Cuevas FJ. Muscle synergies heavily influence the neural control of arm endpoint stiffness and energy consumption. PLOS Comput Biol. (2016) 12(2):e1004737. 10.1371/journal.pcbi.1004737 26867014 PMC4750997

[66] Williams BK Sanders RH Ryu JH Graham-Smith P Sinclair PJ. The kinematic differences between accurate and inaccurate squash forehand drives for athletes of different skill levels. J Sports Sci. (2020) 38(10):1115–23. 10.1080/02640414.2020.1742971 32223529

[67] Yamaguchi T Moriya K Tanabe S Kondo K Otaka Y Tanaka S. Transcranial direct-current stimulation combined with attention increases cortical excitability and improves motor learning in healthy volunteers. J NeuroEngineering Rehabil. (2020) 17(1):23. 10.1186/s12984-020-00665-7 PMC7031972 32075667

[68] de Rugy A Loeb GE Carroll TJ. Muscle coordination is habitual rather than optimal. J Neurosci. (2012) 32(21):7384–91. 10.1523/JNEUROSCI.5792-11.2012 22623684 PMC6622296

[69] Zhou T Wu YH Bartsch A Cuadra C Zatsiorsky VM Latash ML. Anticipatory synergy adjustments: preparing a quick action in an unknown direction. Exp Brain Res. (2013) 226(4):565–73. 10.1007/s00221-013-3469-5 23494385 PMC3634869

[70] Nomura T Kirimoto H. Anodal transcranial direct current stimulation over the supplementary motor area improves anticipatory postural adjustments in older adults. Front Hum Neurosci. (2018) 12:317. 10.3389/fnhum.2018.00317 30123118 PMC6086140

[71] Tamura A Shimura K Inoue Y. Leg and joint stiffness of the supporting leg during side-foot kicking in soccer players with chronic ankle instability. Sports. (2023) 11(11):218. 10.3390/sports11110218 37999435 PMC10674260

[72] Rembrandt HN Riley EA. Evidence of physiological changes associated with single-session pre-frontal tDCS: a pilot study. Front Hum Neurosci. (2025) 19:1549248. 10.3389/fnhum.2025.1549248 40070489 PMC11893991

[73] Haikalis NK Hooyman A Wang P Daliri A Schaefer SY. Placebo effects of transcranial direct current stimulation on motor skill acquisition. Neurosci Lett. (2023) 814:137442. 10.1016/j.neulet.2023.137442 37591359 PMC11101143

[74] Zemková E Pacholek M. Performance in the yo-yo intermittent recovery test may improve with repeated trials: does practice matter? J Funct Morphol Kinesiol. (2023) 8(2):75. 10.3390/jfmk8020075 37367239 PMC10299562

[75] Wiethoff S Hamada M Rothwell JC. Variability in response to transcranial direct current stimulation of the motor cortex. Brain Stimul Basic Transl Clin Res Neuromodulation. (2014) 7(3):468–75. 10.1016/j.brs.2014.02.003 24630848

[76] Hanson NJ Maceri RM Koutakis P. Transcranial direct current stimulation (tDCS) and cycling performance on the 3-minute aerobic test (3mAT): placebo and nocebo effects. Sci Rep. (2024) 14(1):24659. 10.1038/s41598-024-74941-0 39428389 PMC11491469

